# GSDMs are potential therapeutic targets and prognostic biomarkers in clear cell renal cell carcinoma

**DOI:** 10.18632/aging.203973

**Published:** 2022-03-23

**Authors:** Lei Yao, Juanni Li, Zhijie Xu, Yuanliang Yan, Kuan Hu

**Affiliations:** 1 Department of Hepatobiliary Surgery, Xiangya Hospital, Central South University, Changsha 410008, Hunan, China; 2 Department of Pathology, Xiangya Hospital, Central South University, Changsha 410008, Hunan, China; 3 Department of Pharmacy, Xiangya Hospital, Central South University, Changsha 410008, Hunan, China; 4 National Clinical Research Center for Geriatric Disorders, Xiangya Hospital, Central South University, Changsha 410008, Hunan, China

**Keywords:** GSDM family, ccRCC, expression profiles, prognosis, immune infiltration

## Abstract

GSDM family is a group of critical proteins that mediate pyroptosis and plays an important role in cell death and inflammation. However, their specific function in clear cell renal cell carcinoma (ccRCC, KIRC) have not been clarified comprehensively. In this study, we assessed the roles of the GSDM family in expression, prognostic value, functional enrichment analysis, genetic alterations, immune infiltration and DNA methylation in ccRCC patients by using different bioinformatics databases. We found that the expression levels of GADMA-E were significantly higher in ccRCC tissues compared with normal tissues, while the expression level of PJVK was decreased. Moreover, survival analysis indicated that upregulation of GSDME was related to poor overall survival (OS) and recurrence-free survival (RFS) of ccRCC patients. The main function of differentially expressed GSDM homologs was related to ion transport. We also found that the expression profiles of the GSDM family were highly correlated with infiltrating immune cells (i.e., CD8+ T cells, CD4+ T cells, B cells, macrophages, neutrophils and dendritic cells), and there were significant differences in the expression of GSDM family in different ccRCC immune subtypes. Furthermore, DNA methylation analysis indicated that the DNA methylation levels of GSDMA/B/D/E were decreased, while the DNA methylation level of PJVK was increased. In conclusion, this study provides integrated information about abnormal GSDM family members as potential biomarkers for the diagnosis and prognosis of ccRCC. Especially, GSDME was a potential clinical target and prognostic biomarkers for patients with ccRCC.

## INTRODUCTION

Renal cell carcinoma (RCC) is a malignant tumor originating from the renal tubular epithelial system. It is one of the most common urinary tumors in adults [1, 2]. In 2019, there were about 73820 patients with new renal cell carcinoma in the United States, and about 14770 died of renal cell carcinoma [3]. About 70-80% of renal cell carcinoma is clear cell carcinoma (ccRCC), which is the most common pathological subtype of renal cell carcinoma [4]. In recent years, the incidence of renal cell carcinoma worldwide has increased steadily at the rate of 2-4% per year [5]. Surgery is the main treatment for ccRCC because it’s not sensitive to chemotherapy and radiotherapy. Moreover, the prognosis of patients with metastatic clear cell renal carcinoma is very poor, and the five-year survival rate is less than 10% [6–8].

Gasdermins (GSDMs) protein family takes part in diverse functions, which are expressed in a variety of cell types and tissues. GSDMs were firstly found during the positional cloning of mouse skin mutations [9, 10]. It consists of six human genes GSDMA-D, GSDME (DFNA5) and PJVK (DFNB59), all of which encode leucine-rich proteins [9]. Remarkably, among GSDM family proteins, the N-terminal and C-terminal domains are highly conserved. GSDMs are involved in cell death and inflammation, especially the role of GSDMD in inflammatory body signal transduction and pyroptosis. GSDMD is a key factor in pyroptosis because it can be cleaved by inflammatory caspase and form membrane pores [11–14].

Previous studies have demonstrated that, as a key regulator, the GSDM family directly or indirectly affects the development of various tumors like breast cancer [15], gastric cancer [16] and ovarian cancer [17]. Although, the effect of the GSDM family on ccRCC and its mechanism have not been elucidated in detail. Second-generation of sequencing technology and many open source databases provide favorable conditions for us to analyze the GSDM family in an all-round way, which will also help to clarify the role of GSDMs in the development and prognosis of ccRCC.

In present study, a comprehensive bioinformatic analysis was carried out to clarify the effect of GSDM family members in ccRCC. We profoundly evaluated the potential of GSDM family members as therapeutic targets or prognostic biomarkers through the analysis of multiple databases. The aim of this research is to provide more favorable guidance to clinicians in selecting appropriate therapeutic agents and to provide a new basis for survival prediction in ccRCC patients.

## RESULTS

### Abnormal expression of the GDSM family in patients with ccRCC

**Figure 1:**
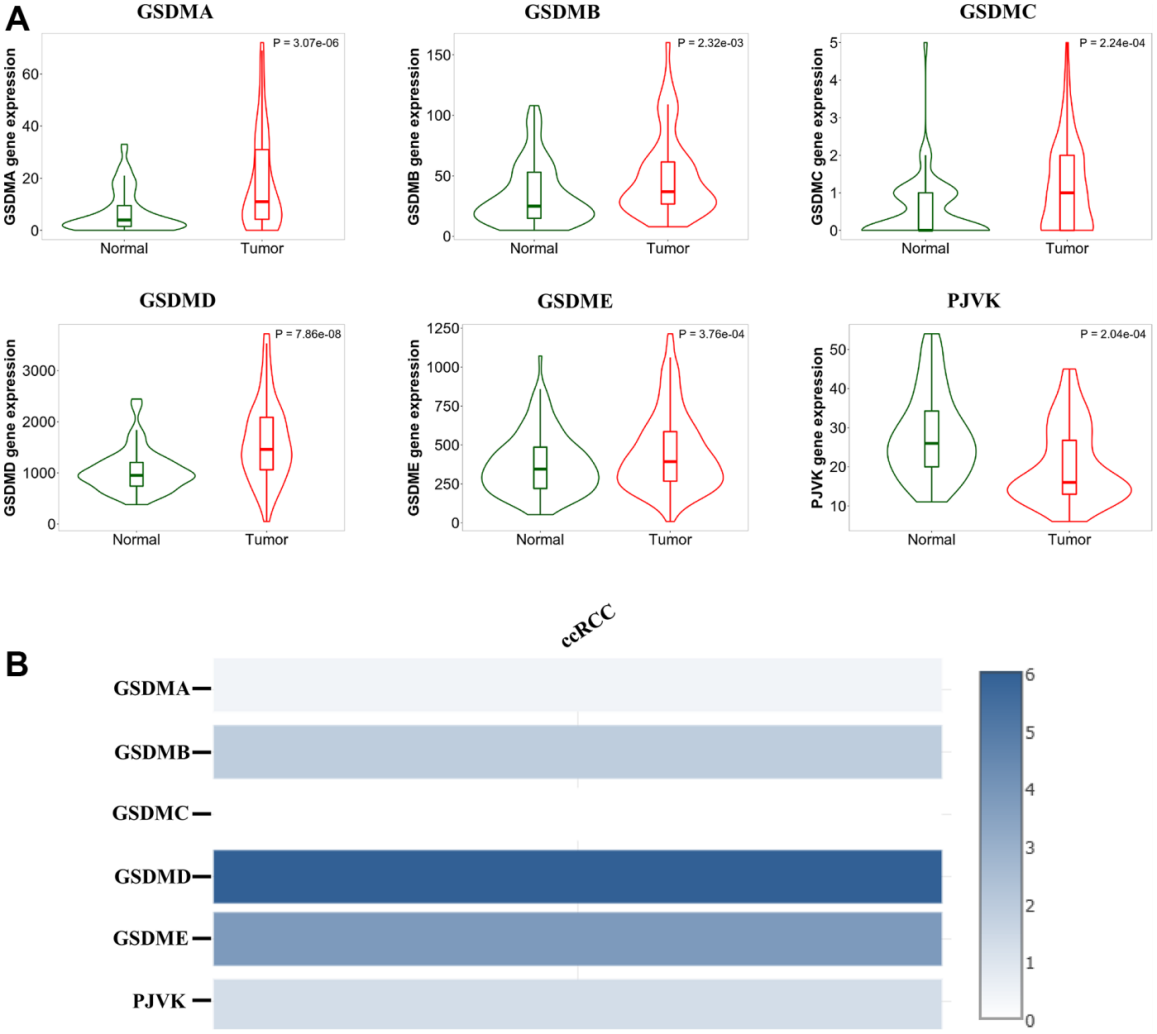
**Analysis of the GSDM family expression levels in ccRCC tissues.** (**A**) The mRNA expression levels of different GSDM family members in ccRCC and normal samples (TNMplot). (**B**) The relative expression of the GSDM family in ccRCC.

First, we explored the mRNA expression levels of the GSDM family (GSDMA-E, PJVK) between ccRCC and normal tissues using the TNMPlot database. The results revealed that GSDMs were expressed at significantly higher levels in ccRCC, except for PJVK, which was expressed at decreased levels in ccRCC tissues (Figure 1A). By comparing the relative expression levels of the GSDMs family in ccRCC though GEPIA2 we found that the expression of GSDMD and GSDME were higher relative to other molecules, and in contrast GSDMC and GADNA were expressed at lower levels (Figure 1B).

**Figure 2:**
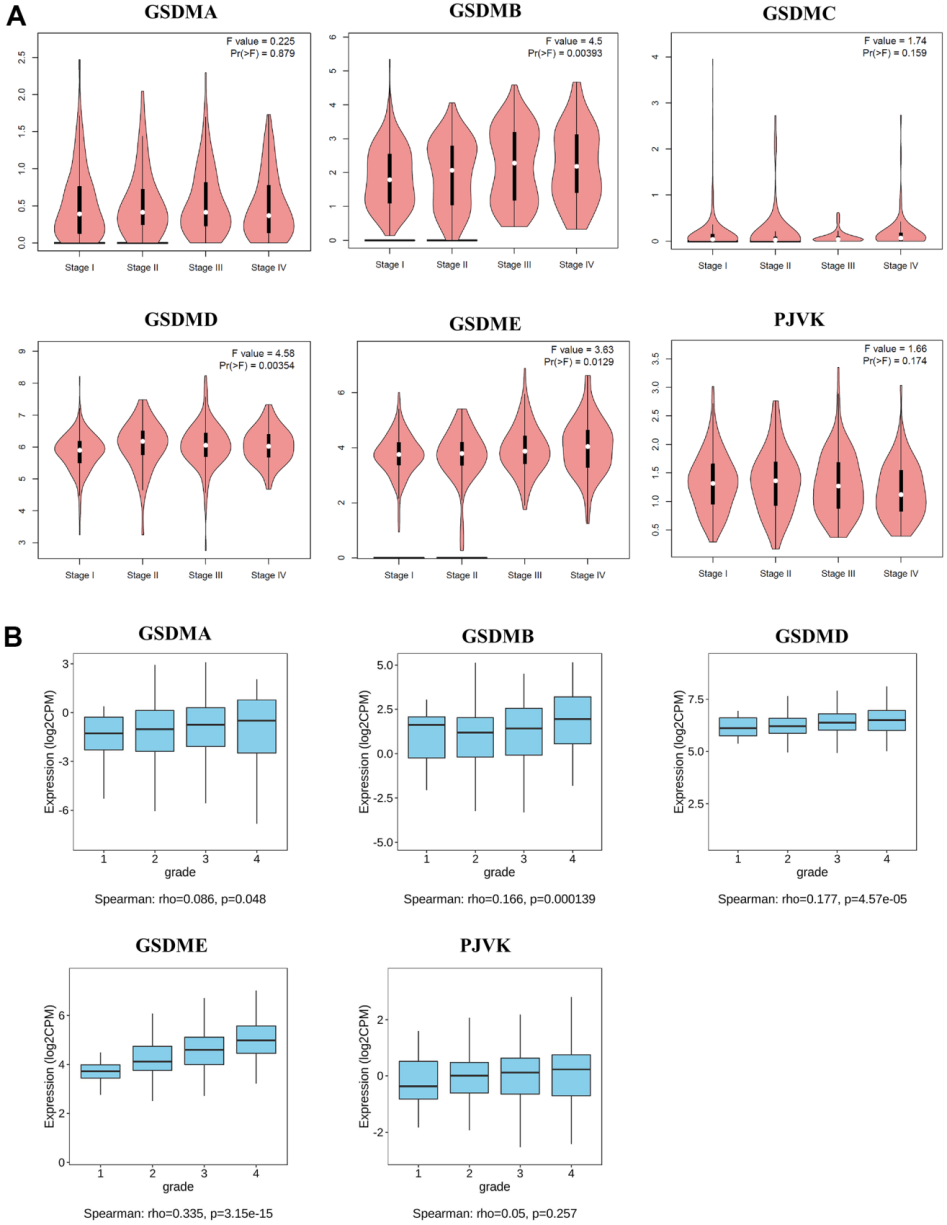
**Relationships between mRNA expression of GSDM family and clinicopathological features of ccRCC.** (**A**) The relationship between mRNA expression and pathological stage of ccRCC patients with different members of the GSDM family (GEPIA2). (**B**) The relationship between mRNA expression and tumor grade of ccRCC patients with different members of the GSDM family (TISIDB). * p < 0.05, ** p < 0.01, *** p < 0.001.

In addition, associations between GSDMs expression and clinicopathological stage and tumor grade of ccRCC were presented by GEPIA2 and TISIDB. Higher mRNA expression levels of GSDMB/D/E were related to higher tumor stage. But the mRNA expression level of PJVK was negatively related to tumor stage, and the higher the tumor stage, the lower its expression level (Figure 2A). Similarly, the mRNA expression levels of GSDMA/B/D/E were positively correlated with tumor grade (Figure 2B).

### Prognostic value of mRNA expression of the GSDM family in ccRCC patients

**Figure 3:**
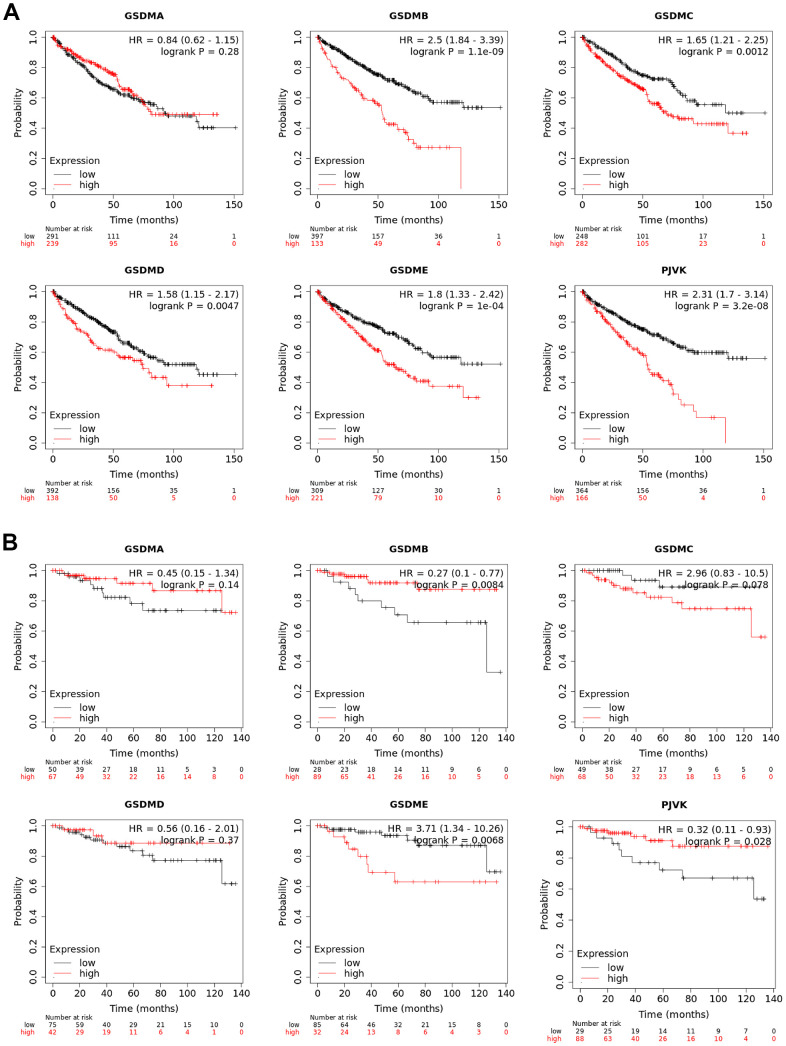
**Prognostic values of GSDM family in ccRCC.** (**A**) The overall survival (OS) curve of GSDM molecules in ccRCC patients (Kaplan-Meier plotter). (**B**) The relapse-free survival (RFS) curve of GSDM molecules in ccRCC patients (Kaplan-Meier plotter).

The prognostic value of different GSDMs expression for ccRCC patients was obtained by Kaplan-Meier analysis, including relapse-free survival (RFS) and overall survival (OS). As shown in the results, higher expression of GSDMB/C/D/E and PJVK showed a significant correlation with poorer OS in ccRCC patients. However, the high transcript expression of GSDMA did not show any correlation (Figure 3A). We then analyzed the relationship between the expression of GSDMs and RFS and showed that the expression of GSDMB/E and PJVK correlated with RFS in ccRCC patients. The transcript levels of GSDME were negatively correlated with RFS in patients, while GSDMB and PJVK showed a positive correlation (Figure 3B). Taken together, GSDME expression predicted both poor OS and RFS, revealing that it may be a promising prognostic biomarker for ccRCC patients.

### Genetic alteration and functional analysis of the GSDM family in ccRCC patients

**Figure 4:**
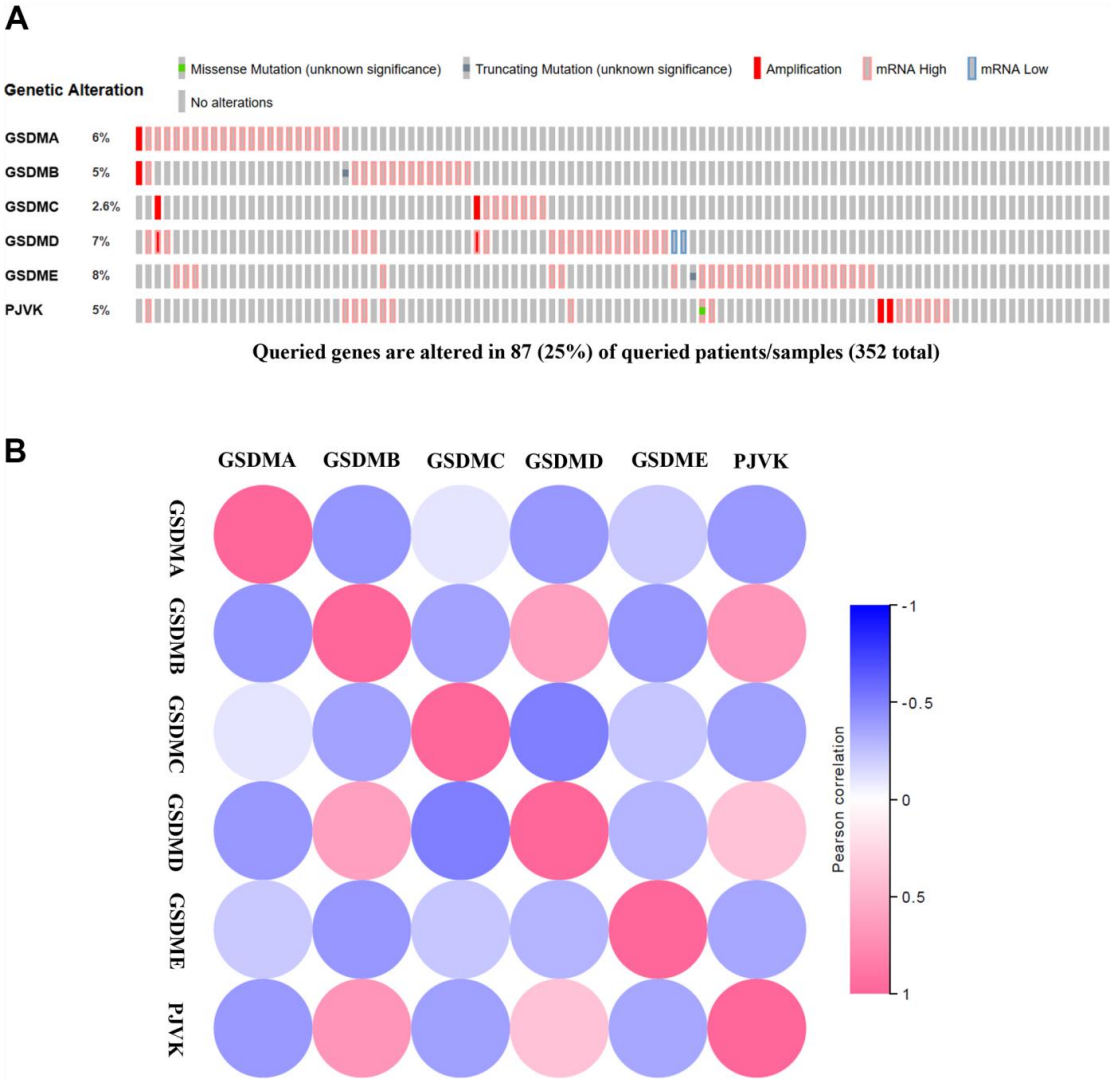
**Genetic alternatives and correlation analysis of the GSDM family in ccRCC.** (**A**) Summary of the alteration rates in each GSDM family member in ccRCC. (**B**) Correlation between different GSDM families in ccRCC (GEPIA).

To further explore the molecular characteristics of the GSDM families that exhibit markedly differential expression, we performed a comprehensive biofunctional analysis of each of them. Differentially expressed GSDM molecules were evaluated by using the TCGA dataset. GSDMA-E and PJVK show altered expression in ccRCC, with 6%, 5%, 2.6%, 7%, 8% and 5% in the alteration rates, respectively (Figure 4A). In the GEPIA2 database, Pearson correction was used to assess the correlation between each family member. We found a strong negative association between GSDMC and GSDMD, GSDMA and GDMB, GSDMB and GSDME. GSDMB was positively correlated with PJVK and GSDMD (Figure 4B).

**Figure 5:**
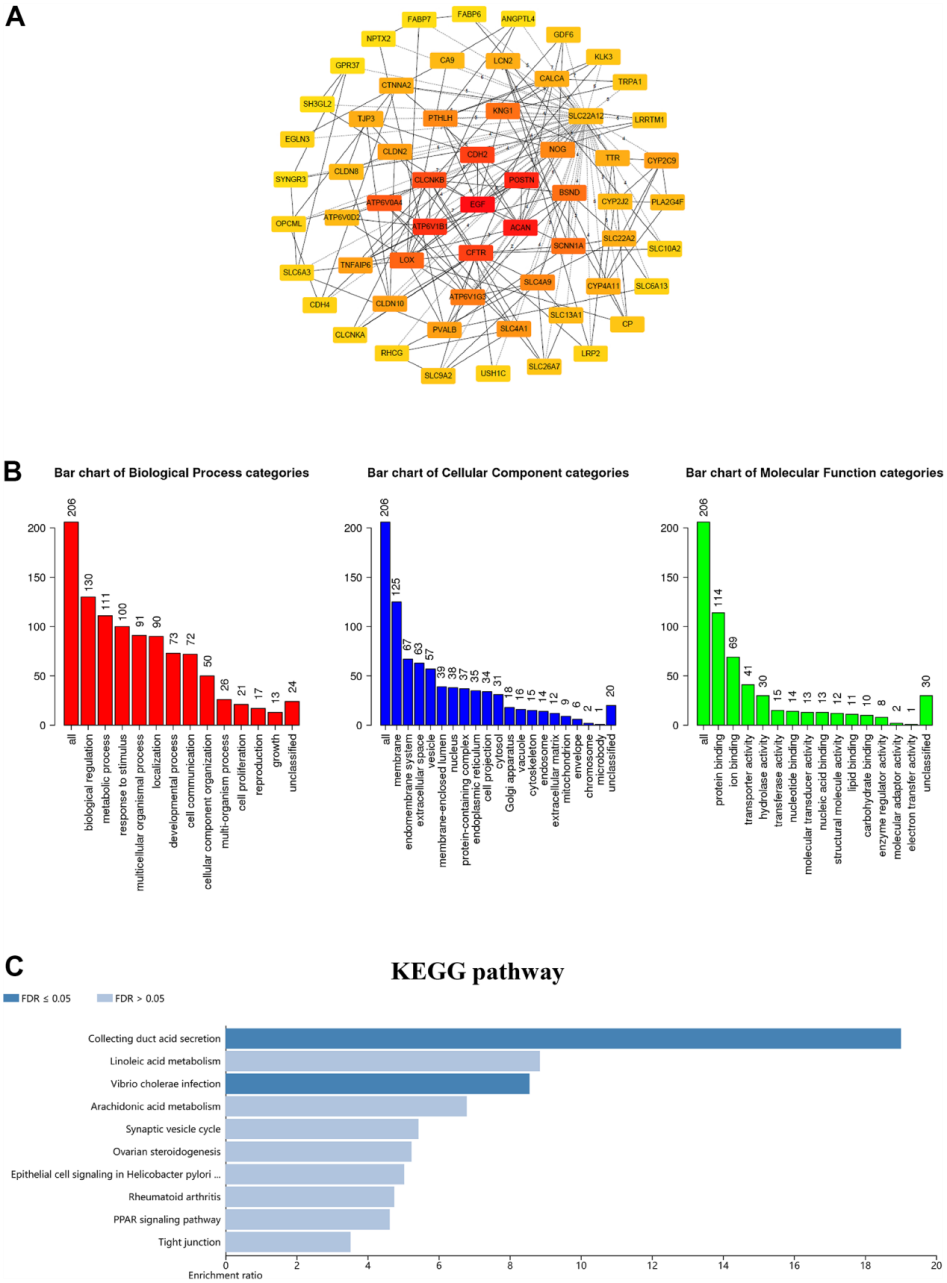
**Coexpression network analysis of the GSDM family in ccRCC.** (**A**) The PPI network of GSDM family interaction partners is generated by the cBioPortal and Cytoscape. (**B**) GO enrichment in biological processes, molecular function and cellular components for the 206 co-expression genes. (**C**) KEGG pathway enrichment analysis of the 206 co-expression genes.

As we know, proteins interact in a variety of ways to form a complex protein-protein interaction (PPI) network that maintains cell activities. Therefore, it becomes crucial to deconstruct the structure and function of protein complexes by constructing PPI patterns. Subsequently, through the cBioPortal database we downloaded 206 differentially expressed molecules with high correlation to GSDM family members to explore the PPI patterns among them and further modified them for visualization using Cytoscape software (Supplementary Table 2). The top 60 genes were shown in Figure 5A. These results suggest that EGF, ACAN, POSTN, ATP6V1B1, CDH2, CFTR, CLCNKB, ATP6V0A4, LOX and BSND as the major molecules are associated with the functional regulation of GSDM family molecules in ccRCC. Go enrichment analysis has annotated the biological significance of differentially expressed biomarkers and their various functions in the organism. KEGG is a genome encyclopedia that used to biologically interpret the fully sequenced genome. In order to explore the biological significance and functions of the above co-expressed molecules, we performed GO annotation and KEGG pathway analysis on them using Webgestalt database. GO analysis confirmed that GSDM family molecules are mainly distributed on cell membranes and play regulatory roles in several important biological processes. Moreover, the results of molecular functional analysis showed that the main function of this family members is to act by binding to different proteins (Figure 5B). KEGG pathway analysis investigated that co-expressed genes were mainly enriched in enriched in collecting duct acid secretion, linoleic acid metabolism and vibrio cholera infection (Figure 5C).

### Immune cell infiltration of GSDM family in ccRCC patients

**Figure 6:**
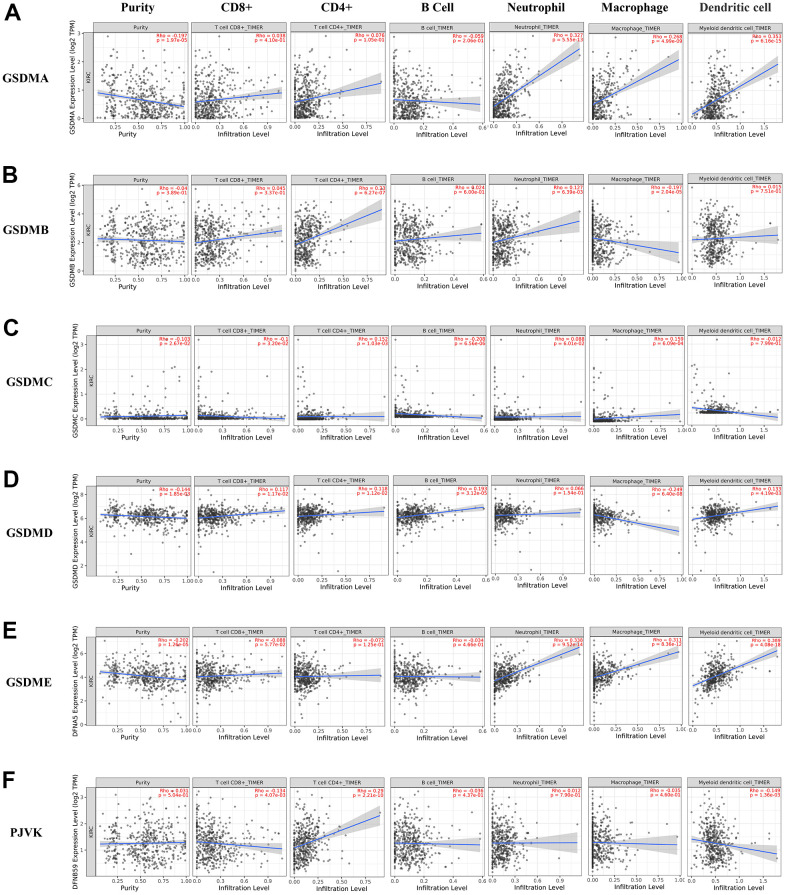
**The correlations between differentially expressed GSDM family and immune cell infiltration.** (**A**–**F**) The effect of GSDMA-E and PJVK on the immune cell infiltration was analyzed by TIMER2.0.

Recently, several findings have revealed that several GSDMs may be involved in inflammatory responses and immune cell infiltration in the microenvironment of a variety of cancers [18, 19]. The associations between individual GSDMs and the infiltration of different immune cells was explored using the TIMER2.0 database. Consequently, the expression of GSDMA and GSDME positively correlated with macrophage, neutrophil and dendritic cell infiltration (Figure 6A, 6E). GSDMB expression was positively related to the infiltration of CD4+ cells and neutrophils, while it was negatively related to macrophages (Figure 6B). GSDMC was negatively related to CD8 + T cells and B cells, and positively related to CD4 + T cells and macrophages (Figure 6C). GSDMD was positively correlated with CD8 + T cells, CD4 + T cells, B cells and dendritic cells, and negatively correlated with macrophages (Figure 6D). We also found that among immune infiltrated cells, the infiltration of CD4+ T cell was positively related to PJVK expression, but the infiltration of CD8+ T cell and dendritic cell was negatively correlated with it (Figure 6F). Furthermore, through the TISIDB website, we explored the role of GSDM family expression among ccRCC immune subtypes. Immune subtypes have been detailed into six types, and we found that GSDMA/B/D and GSDME showed different forms of correlation with different immune subtypes of ccRCC. For instance, GSDME showed high expression in wound healing and TGF-β dominant and low expression in immunologically quiet (Supplementary Figure 1).

### Methylation expression levels of the GSDM family in ccRCC patients

**Figure 7:**
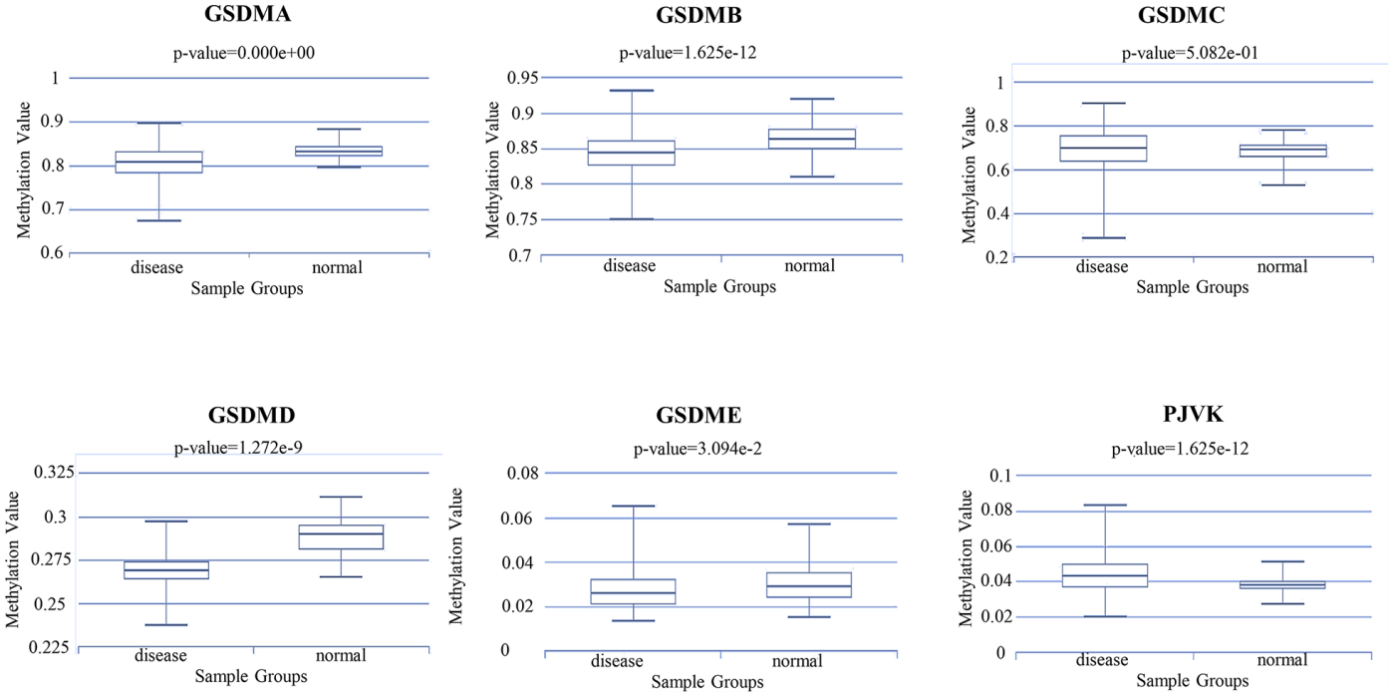
The methylation level of different GSDM family members in ccRCC and normal samples (DiseaseMeth database).

In general, the promoter DNA methylation level is negatively related to the gene expression level under the condition of high gene body methylation [20, 21]. Therefore, it becomes necessary to assess the relationships between DNA methylation and GSDM members expression. Through the DiseaseMeth database, we found that the DNA methylation expression levels of GSDMA/B/D/E in ccRCC samples were significantly lower than those in normal samples. In addition, the DNA methylation expression level of PJVK was higher in ccRCC samples compared to normal samples (Figure 7). Interestingly, previous results confirmed that the expression of GSDMA/B/D/E was significantly elevated in tumor tissues, while PJVK was downregulated. All those suggest that the expression levels of GSDMA/B/D/E and PJVK might be affected by their DNA methylation levels. In addition to DNA methylation, the expression levels of GSDMC may be affected by other regulatory mechanisms.

## DISCUSSION

Cell death is a critical phenomenon of life activities, which is essential for proper homeostatic maintenance and survival in multicellular organisms [22, 23]. Pyroptosis, a form of programmed cell death, which can be mediated by several caspase members [24]. It is mainly divided into classical and non-classical pathways, in which inflammatory bodies play an important role [25]. Moreover, it is believed to be critically involved with the occurrence, invasion and metastasis of tumors [25]. GSDM family (GSDMA-E and PJVK) is a “pore-forming protein” family, with about 45% homologous sequences between their members [26]. GSDMD is the most common and thoroughly studied pyroptosis-related protein known at present, and it is involved in the classical and non-classical pathways of pyroptosis [27]. GSDMB is upregulated in some cancers, like breast cancer, gastric cancer, and colon cancer cells [28]. Therefore, it has been suggested that GSDMB may act as an oncogene to facilitate this process during tumor progression and metastasis [18, 29]. Because sulfide can uniquely bind to the full-length and N-terminal structural domains of GSDMB, GSDMB can induce pyrogenesis [30]. Recently, the function of GSDME in pyroptosis has attracted more and more attention. When GSDME was highly expressed in cells, caspase-3 cleaved GSDME and pyroptosis occurred; When GSDME is low or not expressed, apoptosis occurs [31].

In our research, we all around investigated the expression and clinical prognostic value of the GSDM family in ccRCC, and for the first time, presented the differences in GSDM mRNA expression in tumor tissues of ccRCC patients versus paired adjacent normal tissues. We also found that the GSDM family is closely associated with ccRCC clinicopathological stage and tumor grade. Furthermore, high expression levels of GADMB/C/D/E were negatively correlated with OS in ccRCC patients. In contrast, decreased expression levels of PJVK increased OS time in ccRCC patients. The high expression levels of GSDMB and PJVK were positively correlated with patients' RFS, while the expression levels of GSDME were inversely correlated with patients' RFS. From the above results, we found that only GSDME expression predicted both poor OS and RFS, revealing that it may be a potential prognostic biomarker for ccRCC patients.

During tumorigenesis, multiple genetic alterations at the transcriptional level were often accompanied [32]. The typical mutations of GSDM family mRNA expression level in tumor and normal tissues prove the frequent alterations of genetic information in the process of ccRCC. All those comprehensively prove that the differential expression of the GSDM family may play a key role in ccRCC. Next, Go and KEGG pathway analysis showed that the abnormal expression of GSDM family members in ccRCC mainly affected intracellular ion transport and cell metabolism by regulating the activity of ion channels. By constructing the PPI network of GSDM family members and their 206 co-expressed genes, we found that EGF, ACAN, POSTN, ATP6V1B1, CDH2, CFTR, CLCNKB, ATP6V0A4, LOX and BSND were mainly related to the modulation and function of the GSDM family differentially expressed in ccRCC.

There is growing evidence that tumorigenesis, progression or recurrence may be influenced by immune cell infiltration in the tumor microenvironment and may be an important factor in the efficacy and clinical outcome of immunotherapy [33–35]. Tumor-infiltrating lymphocytes (TILs) in the tumor microenvironment (TME) are independent predictors of prognosis and immunotherapeutic efficacy in many cancers [36, 37]. Our study shows that GSDM family members are closely related to TILs in ccRCC and play an important role in TME. For instance, we demonstrated a significant associations between the GSDMs expression and the infiltration of several immune cells. Previous studies have shown that the RFS of ccRCC patients is related to lower T cell infiltration, lower adaptive immune response and higher neutrophil gene expression [38]. Then, we investigated the expression of the GSDMs in different immune subtypes of ccRCC to investigate the potential mechanism of it. The results reflected the expression of most GSDM molecules differed significantly among the different ccRCC immune subtypes, which may prove that GSDM family members are a promising biomarker for ccRCC diagnosis and participate in immune regulation.

DNA methylation plays an irreplaceable role in the tumorigenesis and development of several tumor types. Our findings have revealed the presence of aberrant expression of DNA methylation in the GSDM family in ccRCC. Meanwhile, other studies have made some breakthroughs in DNA methylation modifications of the GSDM family. Researches have shown the potential of the methylation status of GSDME as a marker for cancer detection in breast and colorectal cancer [39, 40]. Additionally, GSDME methylation status lead to the lower GSDME expression in some tumor cell types, compared with that in normal tissues, which makes it difficult for most tumor cells to activate the pyroptosis [41–43]. Moreover, GSDME methylation was demonstrates to be related with distant metastasis of tumors [44]. In this study, we found that GSDMA/B/D/E methylation levels were significantly lower and PJVK methylation level was significantly higher in ccRCC tissues than those in normal tissues. The methylation status of the GSDMs was consistent with their expression trend, suggesting that DNA methylation may play a critical role in regulating GSDM expression.

Nevertheless, there are a number of limitations of this study. First, there are racial limitations of the cases in the TCGA database, which are mainly white and black, that limited our conclusions for other ethnic populations. Additionally, the molecular mechanisms of action of GSDMs on the occurrence and development of GBM cells are unclear, which need to be further explored. Moreover, although this study has obtained some attractive results, these results are based on the analysis of bioinformatics data, a large number of functional and mechanistic experiments need to be applied to confirm the above conclusions, and large-scale clinical trials are needed to confirm the value of GSDMs for clinical applications.

In this study, we used multiple bioinformatics approaches to construct a comprehensive landscape of GSDM family expression and prognosis in ccRCC. Through comprehensive analysis, we observed that the high expression of GSDME was significantly related to the poor prognosis of ccRCC patients. In addition, the abnormal expression of GSDME was significantly related to the tumor stage and grade in ccRCC. These results provide more favorable guidance to clinicians in selecting appropriate therapeutic agents and to provide a new basis for survival prediction in ccRCC patients.

## MATERIALS AND METHODS

### GEPIA2

GEPIA2 is a web-based TCGA data analysis tool that provides the required functional analysis, such as expression analysis and survival analysis [45]. In this paper, we applied GEPIA2 to explore the expression of genes and the differences in mRNA expression of different genes at different stages of ccRCC (stages 1, 2, 3, and 4). The bioinformatics databases used in this study were summarized in Supplementary Table 1.

### TNMPlot

The TNMplot database [46] is an online analysis tool for comparing gene expression levels in normal, tumor and metastatic tissues. It consists of 57,000 samples and contains several RNA-Seq and microarray datasets, making it the largest transcriptomic cancer database available. In this research, we used TNMPlot to analyze the mRNA expression differences between normal tissues, tumors and metastatic tissues. P < 0.05 were considered statistically significant.

### UALCAN

UALCAN is an interactive web resource based on level 3 RNA sequencing methodology and the clinical data of 31 cancer types from TCGA datasets [47]. In this research, we applied UALCAN to evaluate the mRNA expression level of the GSDM family in ccRCC about nodal metastasis status (N0, 1, 2, 3 and 4). Statistical analyses were performed using Student’s t-test and significant differences were defined as a p-value < 0.05.

### The Human Protein Atlas

The Human Protein Atlas (THPA) is an online database that can be used to validate the expression of target genes using produced antibody and protein expression data [48]. In this research, immunohistochemical images were used to compare the differences in GSDMs protein levels between normal tissues and ccRCC tissues.

### Kaplan-Meier plotter

Kaplan-Meier plotter was used to visualize the prognostic value of the GSDM family in ccRCC [49]. In this research, we investigated the OS/RFS of ccRCC patients visually by K-M survival chart. The following information was provided by the K-M plotter website, including: cases number, median mRNA expression level, HR, 95% CI and p-value.

### Cytoscape

Cytoscape is an open source data curation tool by which high-throughput expression data and other types of data can be integrated into the same network of biomolecular interactions [50]. In this study, we screened 1410 co-expressed molecules of GSDMs from cBioPortal and functionally integrated them by Cytoscape software. The size of the nodes in the network was positively correlated with the strength of the interaction between the proteins.

### WebGestalt

WebGestalt is a comprehensive and interactive web server [51]. In this study, WebGestalt was used for GO enrichment analysis and KEGG pathway analysis.

### TIMER2.0

TIMER2.0 [52] is a web server to comprehensive analysis the relationships between expression patterns and tumor-infiltrating immune cells. TIMER2.0 allows users to enter function-specific parameters and show the resulting map to conveniently access tumor immunological, clinical and genomic characteristics.

### DiseaseMeth2.0

The human disease methylation database (DiseaseMeth2.0) contains not only methylation modifications data, but also annotated data on the DNA methylation status of multiple diseases [53]. We used DiseaseMeth2.0 to detect the possible methylation values of the GSDM family in ccRCC.

### TISIDB

The TISIDB database (http://cis.hku.hk/TISIDB) is an open source website exploring the relationship between genes and tumor immunity interactions, which completed by integrating more than 4176 records from 2530 publications to report 988 genes associated with antitumor immunity [54]. We used the TISIDB database to estimate the mRNA expression level of the GSDM family in different cancer grades (grade 1, 2, 3 and 4) of ccRCC, and analyzed GSDM family expression in different immune subtypes.

## Supplementary Material

Supplementary Figure 1

Supplementary Table 1

Supplementary Table 2
