# Supplementary material for: GSDMs are potential therapeutic targets and prognostic biomarkers in clear cell renal cell carcinoma
Source: Aging (Albany NY). 2022 Mar 23;14(6):2758–74. doi: 10.18632/aging.203973 (PMC9004560; doi:10.18632/aging.203973)
Supplement: Supplementary Figure 1 [file aging-14-203973-s001.pdf]

SUPPLEMENTARY FIGURE

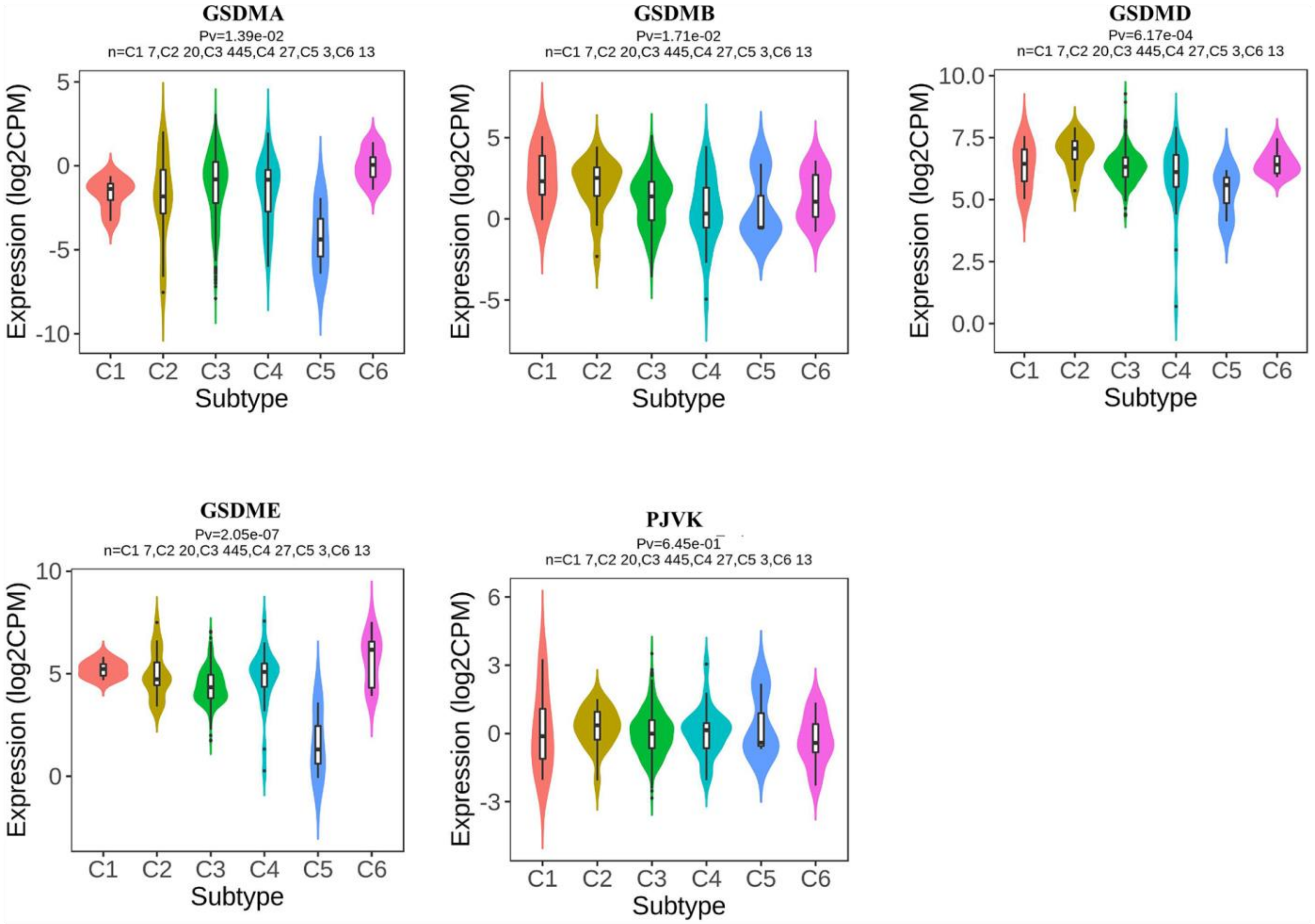

Supplementary Figure 1. The relationship between GSDM molecules' expression and ccRCC immune subtypes, including wound healing (C1), IFN-gamma dominant (C2), inflammatory (C3), lymphocyte depleted (C4), immunologically quiet (C5) and TGF-β dominant (C6) (TISIDB).
