# Supplementary material for: GSDMs are potential therapeutic targets and prognostic biomarkers in clear cell renal cell carcinoma
Source: Aging (Albany NY). 2022 Mar 23;14(6):2758–74. doi: 10.18632/aging.203973 (PMC9004560; doi:10.18632/aging.203973)
Supplement: Supplementary Table 1 [file aging-14-203973-s002.pdf]

SUPPLEMENTARY TABLE

Supplementary Table 1. A series of bioinformatics databases for analyzing the role of the GSDM family in the biological processes of ccRCC.

Databases	Authors	Samples	Homepage links
GEPIA2	Tang Z. et al.	Tissues	http://gepia.cancer-pku.cn/
UALCAN	Chandrashekar DS	Tissues	http://ualcan.path.uab.edu/index.html
TNMPlot	Bartha Á, Győrffy B	Tissues	https://www.tnmplot.com/
Kaplan-Meier plotter	Gyorffy B. et al.	Tissues	http://kmplot.com/analysis/
The Human Protein Atlas	Anna Asplund. et al.	Tissues	https://www.proteinatlas.org/
cBioPortal	Cerami E. et al.	Tissues	http://www.cbioportal.org/
Cytoscape	Doncheva NT et al.	-	-
WebGestalt	Liao Y. et al.	-	http://webgestalt.org/
TIMER2.0	Li T. et al.	Tissues	https://cistrome.shinyapps.io/timer/
TISIDB	Ru B. et al	Tissues	http://cis.hku.hk/TISIDB/
DiseaseMeth 2.0	Xiong Y. et al.	Tissues	http://bio-bigdata.hrbmu.edu.cn/diseasemeth/
