# Supplementary material for: GSDMs are potential therapeutic targets and prognostic biomarkers in clear cell renal cell carcinoma
Source: Aging (Albany NY). 2022 Mar 23;14(6):2758–74. doi: 10.18632/aging.203973 (PMC9004560; doi:10.18632/aging.203973)
Supplement: Supplementary Table 2 [file aging-14-203973-s003.docx]

**Supplementary Table 2. The GSDM family member-associated co-expressed genes in ccRCC.**

| Gene | Log Ratio | p-Value | Higher expression in |
| --- | --- | --- | --- |
| MYH14 | 1.69 | 1.86E-06 | Altered group |
| FABP7 | -4.27 | 6.54E-06 | Unaltered group |
| CCDC78 | 1.66 | 1.52E-05 | Altered group |
| GPR37 | 1.67 | 9.83E-05 | Altered group |
| CLEC18A | -2.03 | 1.01E-04 | Unaltered group |
| ASPG | -2.5 | 1.23E-04 | Unaltered group |
| STK33 | 1.83 | 1.43E-04 | Altered group |
| STC2 | -1.6 | 1.98E-04 | Unaltered group |
| DEFB1 | 2.06 | 2.45E-04 | Altered group |
| CLEC18B | -2.16 | 3.19E-04 | Unaltered group |
| CDCA2 | -1.71 | 3.21E-04 | Unaltered group |
| SMOC1 | 2.54 | 3.25E-04 | Altered group |
| VWCE | -1.9 | 4.07E-04 | Unaltered group |
| TFCP2L1 | 2.16 | 4.08E-04 | Altered group |
| NR2E1 | -1.51 | 4.73E-04 | Unaltered group |
| SLC6A3 | -3.43 | 4.81E-04 | Unaltered group |
| MTCL1 | -1.59 | 4.83E-04 | Unaltered group |
| NOG | -1.52 | 4.85E-04 | Unaltered group |
| SEMA5B | -1.91 | 6.41E-04 | Unaltered group |
| TNFAIP6 | -2.18 | 6.85E-04 | Unaltered group |
| POSTN | -1.61 | 7.30E-04 | Unaltered group |
| GDF6 | -1.56 | 7.97E-04 | Unaltered group |
| OPCML | -1.79 | 8.06E-04 | Unaltered group |
| RHBG | 2.81 | 8.08E-04 | Altered group |
| COL21A1 | -1.6 | 8.22E-04 | Unaltered group |
| LINC01587 | -1.53 | 8.72E-04 | Unaltered group |
| SYNGR3 | 1.87 | 8.76E-04 | Altered group |
| HAGHL | 1.85 | 8.94E-04 | Altered group |
| ANGPTL4 | -2.39 | 9.63E-04 | Unaltered group |
| LINC01018 | 1.68 | 9.71E-04 | Altered group |
| GRIK3 | -1.83 | 9.96E-04 | Unaltered group |
| MYO3A | -1.78 | 1.10E-03 | Unaltered group |
| TJP3 | 2.01 | 1.11E-03 | Altered group |
| CLEC18C | -2.08 | 1.15E-03 | Unaltered group |
| KLK4 | 2.76 | 1.15E-03 | Altered group |
| SLC5A8 | -2.74 | 1.21E-03 | Unaltered group |
| INPP5J | 2.21 | 1.23E-03 | Altered group |
| RIC3 | 1.51 | 1.24E-03 | Altered group |
| ANO5 | 1.86 | 1.40E-03 | Altered group |
| SLC17A4 | -2.18 | 1.46E-03 | Unaltered group |
| DIRAS2 | -1.76 | 1.54E-03 | Unaltered group |
| ACAN | -1.63 | 1.56E-03 | Unaltered group |
| ADORA2A-AS1 | -1.53 | 1.58E-03 | Unaltered group |
| SYNE4 | 1.69 | 1.60E-03 | Altered group |
| TGFA | -1.64 | 1.61E-03 | Unaltered group |
| CA9 | -2.97 | 1.69E-03 | Unaltered group |
| PPM1E | 1.56 | 1.72E-03 | Altered group |
| HCN2 | 2.04 | 1.73E-03 | Altered group |
| LIPH | 1.56 | 1.74E-03 | Altered group |
| GSDMC | 1.54 | 1.81E-03 | Altered group |
| DNASE1 | 1.68 | 1.81E-03 | Altered group |
| LINC01559 | -1.8 | 1.81E-03 | Unaltered group |
| CYP11A1 | 2.11 | 1.82E-03 | Altered group |
| EGLN3 | -1.85 | 1.91E-03 | Unaltered group |
| AVPR1B | -1.53 | 2.01E-03 | Unaltered group |
| GCGR | 2.47 | 2.07E-03 | Altered group |
| TMPRSS2 | 2.41 | 2.11E-03 | Altered group |
| NR0B2 | 2.52 | 2.22E-03 | Altered group |
| SLC5A12 | -2.41 | 2.28E-03 | Unaltered group |
| GALNT17 | 2.36 | 2.32E-03 | Altered group |
| SLITRK5 | -1.58 | 2.35E-03 | Unaltered group |
| ENPP3 | -2.42 | 2.48E-03 | Unaltered group |
| SLC6A18 | -1.9 | 2.52E-03 | Unaltered group |
| SMIM5 | 1.68 | 2.74E-03 | Altered group |
| LGI4 | -1.73 | 2.85E-03 | Unaltered group |
| SLC26A7 | 2.46 | 2.88E-03 | Altered group |
| EGF | 1.56 | 2.90E-03 | Altered group |
| ESRP1 | 2.06 | 2.91E-03 | Altered group |
| FA2H | 1.69 | 2.96E-03 | Altered group |
| CTNNA2 | 1.97 | 3.11E-03 | Altered group |
| CLCNKA | 2.28 | 3.13E-03 | Altered group |
| RASAL1 | -1.58 | 3.18E-03 | Unaltered group |
| TRIM50 | 2.28 | 3.32E-03 | Altered group |
| COL23A1 | -2.26 | 3.40E-03 | Unaltered group |
| C1QL4 | -1.66 | 3.49E-03 | Unaltered group |
| RHCG | 3.35 | 3.50E-03 | Altered group |
| SLC10A2 | -1.99 | 3.51E-03 | Unaltered group |
| CDHR1 | -1.7 | 3.52E-03 | Unaltered group |
| ALPK2 | -2.07 | 3.67E-03 | Unaltered group |
| DUSP15 | 1.77 | 3.69E-03 | Altered group |
| DMRT2 | 2.63 | 3.71E-03 | Altered group |
| IRX3 | -1.57 | 3.83E-03 | Unaltered group |
| KISS1R | -1.86 | 3.84E-03 | Unaltered group |
| ATP6V0D2 | 2.91 | 3.90E-03 | Altered group |
| DIRAS1 | 1.69 | 3.92E-03 | Altered group |
| KLK15 | 2.18 | 4.12E-03 | Altered group |
| CLCNKB | 2.69 | 4.17E-03 | Altered group |
| EPB41L4B | 1.7 | 4.22E-03 | Altered group |
| PVALB | 2.95 | 4.28E-03 | Altered group |
| PRIMA1 | -2.36 | 4.36E-03 | Unaltered group |
| TMEM61 | 1.96 | 4.38E-03 | Altered group |
| FOXI1 | 3 | 4.53E-03 | Altered group |
| KLK1 | 2.93 | 4.59E-03 | Altered group |
| CYP2J2 | -2.27 | 4.63E-03 | Unaltered group |
| PART1 | 2.37 | 4.67E-03 | Altered group |
| FAM83F | 1.77 | 4.70E-03 | Altered group |
| FBN3 | 2.17 | 4.74E-03 | Altered group |
| PRUNE2 | -1.71 | 4.98E-03 | Unaltered group |
| FGF9 | 2.08 | 5.01E-03 | Altered group |
| SCNN1A | 1.7 | 5.04E-03 | Altered group |
| NDUFA4L2 | -1.86 | 5.06E-03 | Unaltered group |
| ALKAL2 | -1.71 | 5.17E-03 | Unaltered group |
| SH3GL2 | 1.84 | 5.22E-03 | Altered group |
| TRPA1 | -1.68 | 5.27E-03 | Unaltered group |
| NAT8 | -1.91 | 5.32E-03 | Unaltered group |
| STAP1 | 2.13 | 5.34E-03 | Altered group |
| CUBN | -1.88 | 5.34E-03 | Unaltered group |
| C4BPB | 1.75 | 5.41E-03 | Altered group |
| TMEM213 | 2.78 | 5.46E-03 | Altered group |
| SORCS3 | -2.03 | 5.48E-03 | Unaltered group |
| MAL | 1.72 | 5.53E-03 | Altered group |
| ATP6V0A4 | 2.95 | 5.59E-03 | Altered group |
| PLA2G4F | 2.4 | 5.61E-03 | Altered group |
| SPINK7 | 1.59 | 5.63E-03 | Altered group |
| MCOLN3 | 1.54 | 5.66E-03 | Altered group |
| CCL20 | -1.6 | 5.66E-03 | Unaltered group |
| ASB5 | 1.7 | 5.69E-03 | Altered group |
| SHOC1 | 1.52 | 5.81E-03 | Altered group |
| ADGRF1 | 2.13 | 5.82E-03 | Altered group |
| PTHLH | -1.59 | 5.90E-03 | Unaltered group |
| CKMT1B | 2.15 | 5.97E-03 | Altered group |
| CLDN10 | -1.64 | 6.14E-03 | Unaltered group |
| LCN2 | 1.97 | 6.22E-03 | Altered group |
| LRRTM1 | 1.85 | 6.35E-03 | Altered group |
| UPB1 | -1.75 | 6.43E-03 | Unaltered group |
| HEPACAM2 | 2.71 | 6.53E-03 | Altered group |
| SLC7A9 | -1.57 | 6.65E-03 | Unaltered group |
| PNMA2 | -1.58 | 6.87E-03 | Unaltered group |
| MAP7D2 | -1.83 | 6.92E-03 | Unaltered group |
| AQP6 | 2.36 | 6.95E-03 | Altered group |
| THRSP | 1.64 | 7.14E-03 | Altered group |
| SLC38A3 | 1.63 | 7.15E-03 | Altered group |
| ATP6V1B1 | 2.28 | 7.17E-03 | Altered group |
| SLC9A2 | 1.96 | 7.27E-03 | Altered group |
| A1CF | -1.6 | 7.45E-03 | Unaltered group |
| KLK3 | 1.67 | 7.52E-03 | Altered group |
| SYT10 | 1.66 | 7.59E-03 | Altered group |
| GPRC6A | 1.82 | 7.61E-03 | Altered group |
| ATP6V1G3 | 2.51 | 7.70E-03 | Altered group |
| EDN2 | -1.54 | 7.90E-03 | Unaltered group |
| RNF186 | -1.66 | 7.92E-03 | Unaltered group |
| CLGN | 1.53 | 7.93E-03 | Altered group |
| CWH43 | 2.01 | 7.98E-03 | Altered group |
| PCSK6 | -1.51 | 8.41E-03 | Unaltered group |
| TMEM255A | 1.73 | 8.47E-03 | Altered group |
| GALNT14 | -1.73 | 8.48E-03 | Unaltered group |
| INSYN1 | 1.6 | 8.52E-03 | Altered group |
| TEX15 | -1.66 | 8.90E-03 | Unaltered group |
| TRIM15 | -1.57 | 9.20E-03 | Unaltered group |
| ERP27 | 1.51 | 9.28E-03 | Altered group |
| CDH4 | -1.56 | 9.44E-03 | Unaltered group |
| HHATL | 2.16 | 9.60E-03 | Altered group |
| CNTN3 | 1.66 | 9.73E-03 | Altered group |
| DNAH11 | -1.84 | 9.75E-03 | Unaltered group |
| CLDN2 | -1.66 | 9.79E-03 | Unaltered group |
| NMRK2 | 2.1 | 0.01 | Altered group |
| FABP6 | -1.65 | 0.0103 | Unaltered group |
| BBOX1 | -1.73 | 0.0104 | Unaltered group |
| KCNK3 | -1.62 | 0.0105 | Unaltered group |
| BSND | 2.19 | 0.0107 | Altered group |
| LOX | -1.58 | 0.0108 | Unaltered group |
| EPO | -1.56 | 0.0108 | Unaltered group |
| CFTR | 2.48 | 0.0109 | Altered group |
| ADH1C | 1.56 | 0.0111 | Altered group |
| MOG | 1.55 | 0.0112 | Altered group |
| CKMT1A | 1.95 | 0.0114 | Altered group |
| KRTAP5-8 | 1.5 | 0.0115 | Altered group |
| CP | -2.14 | 0.0117 | Unaltered group |
| PIP | 1.62 | 0.0118 | Altered group |
| CDH2 | -1.65 | 0.0118 | Unaltered group |
| SLC4A9 | 1.67 | 0.012 | Altered group |
| SCG3 | 1.69 | 0.012 | Altered group |
| SCGN | -1.84 | 0.0121 | Unaltered group |
| SLC5A10 | -1.6 | 0.0124 | Unaltered group |
| KBTBD12 | 1.54 | 0.0133 | Altered group |
| MCCD1 | 1.53 | 0.0137 | Altered group |
| SLC28A1 | -1.73 | 0.0139 | Unaltered group |
| EPN3 | 1.55 | 0.014 | Altered group |
| LRP2 | -1.97 | 0.014 | Unaltered group |
| PSG4 | 1.82 | 0.0144 | Altered group |
| CLDN8 | 2.37 | 0.0147 | Altered group |
| TTR | 2 | 0.015 | Altered group |
| EPHA7 | -1.62 | 0.0156 | Unaltered group |
| CYP2C9 | 1.51 | 0.016 | Altered group |
| SLC22A2 | -1.88 | 0.0161 | Unaltered group |
| LINC00887 | -1.66 | 0.0166 | Unaltered group |
| CLNK | 1.79 | 0.0168 | Altered group |
| PNCK | -1.73 | 0.0175 | Unaltered group |
| NUPR2 | 1.66 | 0.0176 | Altered group |
| SLC2A5 | -1.57 | 0.0195 | Unaltered group |
| PSG5 | 1.61 | 0.0196 | Altered group |
| NPTX2 | -1.53 | 0.02 | Unaltered group |
| CALCA | 1.8 | 0.0204 | Altered group |
| SLC4A1 | 2.07 | 0.0215 | Altered group |
| IGFBP1 | 1.88 | 0.0229 | Altered group |
| SLC17A3 | -1.94 | 0.0233 | Unaltered group |
| USH1C | -1.61 | 0.0246 | Unaltered group |
| GGT1 | -1.52 | 0.0263 | Unaltered group |
| SLC13A1 | -1.75 | 0.0271 | Unaltered group |
| KNG1 | 1.65 | 0.0284 | Altered group |
| SLC6A13 | -1.52 | 0.0306 | Unaltered group |
| CD70 | -1.61 | 0.0311 | Unaltered group |
| AOC1 | -1.57 | 0.0359 | Unaltered group |
| SLC22A12 | -1.56 | 0.0371 | Unaltered group |
| SFTPB | 1.72 | 0.0396 | Altered group |
| CYP4A11 | -1.82 | 0.0445 | Unaltered group |
